# Association of Dipeptidyl Peptidase-4 Inhibitor Use with COVID-19 Mortality in Diabetic Patients: A Nationwide Cohort Study in Korea

**DOI:** 10.3390/jcm14165815

**Published:** 2025-08-17

**Authors:** Jung Wan Park, Mi Kyung Kwak, Samel Park, Nam Hun Heo, Eun Young Lee

**Affiliations:** 1 Division of Infectious Diseases, Department of Internal Medicine, Soonchunhyang University Cheonan Hospital, College of Medicine, Soongchunhyang University, Cheonan 31151, Republic of Korea; splendidmagic@schmc.ac.kr; 2 Department of Internal Medicine, Hallym University Dongtan Sacred Heart Hospital, Dongtan 18450, Republic of Korea; creamtea38@gmail.com; 3 Division of Nephrology, Department of Internal Medicine, Soonchunhyang University Cheonan Hospital, Soonchunhyang University College of Medicine, Cheonan 31151, Republic of Korea; samelpark17@schmc.ac.kr; 4 Clinical Trial Center, Soonchunhyang University Hospital, Cheonan 31151, Republic of Korea; hello3933@schmc.ac.kr

**Keywords:** DPP-4 inhibitors, COVID-19, diabetes mellitus

## Abstract

**Background/Objectives:** Patients with diabetes mellitus face increased risk of severe outcomes and mortality from COVID-19. Dipeptidyl peptidase-4 (DPP-4) inhibitors, widely used antidiabetic agents, are hypothesized to affect COVID-19 outcomes via anti-inflammatory and immune-modulating mechanisms. However, real-world evidence, especially in Korean populations, remains limited. **Methods:** We conducted a retrospective cohort study using Korea’s nationwide Health Insurance Review and Assessment (HIRA) database. Adults with diabetes hospitalized for confirmed COVID-19 between 1 March 2021, and 28 February 2022, were included and stratified by DPP-4 inhibitor use. The primary outcome was 30-day all-cause mortality. Cox proportional hazards models adjusted for age, sex, and comorbidities estimated hazard ratios (HRs). Subgroup analyses examined angiotensin receptor blocker (ARB) and insulin use. **Results:** Among 16,134 eligible patients, 7082 received DPP-4 inhibitors. The 30-day mortality rate was lower in DPP-4 inhibitor users than non-users (4.3% vs. 10.3%, *p* < 0.0001). Adjusted analyses showed DPP-4 inhibitor use was associated with reduced mortality risk (adjusted HR: 0.455; 95% CI: 0.414–0.499). Subgroup analyses yielded consistent results across ARB and insulin users. Kaplan-Meier curves demonstrated higher survival probability in the DPP-4 inhibitor group. **Conclusions:** In this nationwide Korean cohort, DPP-4 inhibitor use was associated with lower mortality among hospitalized diabetic patients with COVID-19. While these findings suggest a potential benefit, causality cannot be confirmed due to the observational design. Prospective studies are needed to verify these associations and explore underlying mechanisms.

## 1. Introduction

Over the past four years, we have experienced an unprecedented COVID-19 pandemic. In the meantime, as of 12 May 2024, there have been over 775 million confirmed cases and more than 7 million patient deaths [1]. Diabetes is one of the most significant risk factors for increased mortality in COVID-19, as documented in numerous studies [2]. Several studies have explored the effect of diabetes medications on COVID-19 [3,4,5,6]. Additionally, the impact of glycemic control on COVID-19 mortality has been studied. A meta-analysis found that patients who continued oral antidiabetic drugs had lower mortality rates and reduced inflammation markers, emphasizing the importance of continuing these medications to improve blood sugar control and reduce mortality in diabetic patients [7]. Among them, medications such as dipeptidyl peptidase-4 (DPP-4) inhibitors are particularly noted for their association with the angiotensin-converting enzyme (ACE) [8,9]. Given that SARS-CoV-2 infects humans via the ACE2 receptor, it is plausible to hypothesize that DPP-4 inhibitors may have a greater impact on COVID-19 mortality than other agents [10]. Several systematic reviews and meta-analyses have reported mixed results regarding the association between DPP-4 inhibitor use and COVID-19 outcomes. While some meta-analyses observed mortality risk reductions with DPP-4 inhibitor use [11,12], others found neutral effects or even increased risks for hospitalization or intensive care unit (ICU) admission (e.g., ~1.5× higher risk) [13]. Additionally, narrative reviews have proposed plausible non-glycemic mechanisms, including inhibition of viral entry, anti-inflammatory effects, and attenuation of fibrotic pathways, through which DPP-4 inhibitors might influence the COVID-19 disease course beyond glycemic control [14]. Despite these mechanistic hypotheses, large-scale randomized controlled trials remain scarce, and observational evidence is often inconclusive due to heterogeneity in populations and varying adjustment for confounders [15]. However, these studies lacked data from Korean populations, and we aimed to analyze Korean data to evaluate the impact of DPP-4 inhibitors on real-world COVID-19 mortality within this group. Focusing on the Korean population is particularly important due to several unique factors. Korea has one of the fastest aging populations globally, with nearly 18% aged ≥65 years, potentially increasing severe COVID-19 risk in diabetic patients. Its universal single-payer healthcare system ensures equitable access to testing and hospitalization, reducing disparities seen elsewhere. During the study period, Korea’s epidemiological profile was marked by high prevalence of Delta and early Omicron variants, differing from patterns in Western populations. These demographic, healthcare, and virological factors highlight the need for population-specific analyses to guide tailored treatments and public health policies. To address this evidence gap, we conducted a nationwide cohort study using the Korean Health Insurance Review and Assessment (HIRA) database, which offers comprehensive clinical and treatment information for nearly the entire Korean population. This study is the first of its kind in South Korea and one of the few globally to incorporate real-world inpatient data, along with detailed comorbidity and medication histories. Through additional subgroup analyses by insulin and ARB use, we aimed to refine our understanding of the potential mortality-reducing effects of DPP-4 inhibitors in diabetic patients with COVID-19.

## 2. Materials and Methods

### 2.1. Data Sources and Record Linkage

We analyzed the HIRA database of patients diagnosed with diabetes mellitus (DM) and receiving DM medications who were hospitalized and treated for COVID-19 between 1 March 2021, and 28 February 2022. Through the HIRA service, we identified the patients’ gender, age, and type of medical institutions where the patients were admitted, days of hospitalization, oxygen prescription, death status, whether the patient required ICU admission during hospitalization, and underlying medical conditions (hypertension, hyperlipidemia, chronic renal failure, ischemic heart disease, stroke, congestive heart failure, etc.). Drug information included brand and generic names, prescription dates, and duration and route of administration. Diagnoses were coded according to the International Classification of Diseases (10th revision; ICD-10).

Our study was approved by the Institutional Review Board (IRB) of Soonchunhyang University Cheonan Hospital (Cheonan, Korea) (IRB No. 2022-08-059). The requirement for patient informed consent was waived, as the study involved secondary analysis of anonymized administrative data.

### 2.2. Study Population (Case Definition)

For this study, we used the case definition of patients aged 19 years or older with confirmed COVID-19 who also had diabetes.

We defined patients with confirmed COVID-19 as those assigned the disease code U07.1 (Coronavirus disease 2019 with confirmed virus). Additionally, we defined patients with diabetes as those with at least one of the disease codes E10 through E14, or with at least one prescription for a diabetes medication.

Among the patients’ underlying disease information, hypertension was defined as those with disease codes I10–13 and 15, hyperlipidemia as E78, chronic renal failure as N18 and 19, chronic obstructive pulmonary disease as J41–44, ischemic heart disease as I20–25, stroke as I21–22, and congestive heart failure as I150. Specifically, patients with chronic kidney disease who are on dialysis were categorized as those with disease codes V001, V003, or V005.

### 2.3. Statistical Analysis

Statistical analyses were conducted to compare baseline characteristics between DPP-4 inhibitor users and non-users. Continuous variables were analyzed using Student’s *t*-test or Mann–Whitney U test, and categorical variables using Chi-square or Fisher’s exact test.

To address potential confounding, multivariable Cox proportional hazards models were used to estimate adjusted hazard ratios (HRs) for 30-day mortality. Variables such as age, sex, hypertension, hyperlipidemia, chronic kidney disease, ischemic heart disease, and chronic obstructive pulmonary disease were selected a priori based on clinical relevance and included as covariates in the models. Hospitalization indicators (e.g., oxygen therapy, ICU admission) served as proxies for baseline severity, and exclusion criteria (e.g., outpatient-only cases, hospital stays >30 days) ensured comparability. Stepwise selection refined the model, and sensitivity analysis using a fully adjusted model with all pre-specified covariates yielded consistent results, supporting robustness.

In SAS (version 9.3; SAS Institute Inc., Cary, NC, USA), PROC LIFETEST generated survival curves and evaluated between-group differences using the log-rank test. Kaplan-Meier curves were then re-visualized in R (version 4.3.0) using the survfit function from the survival package.

To further validate robustness, sensitivity analyses were conducted using propensity score matching (PSM) and inverse probability of treatment weighting (IPTW) based on age, sex, and comorbidities. Covariate balance was confirmed (standardized mean difference <0.1), and matched or weighted Cox models consistently showed significant associations between DPP-4 inhibitor use and reduced mortality.

## 3. Results

### 3.1. Baseline Characteristics

**Figure 1:**
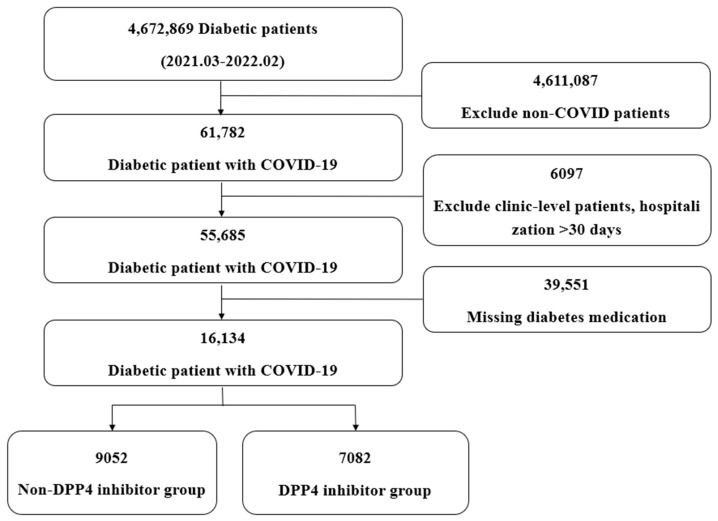
Patient selection and exclusion flowchart illustrating inclusion and exclusion criteria and final cohort size.

During the study screening period, 4,672,869 patients with diabetes were identified, of whom 61,782 had co-morbid COVID-19. We excluded cases treated at a primary care institution, cases with only outpatient treatment records, cases with hospitalizations longer than 30 days, cases with missing information on diabetes medications, and cases with unclear information on diabetes medication prescriptions. These exclusion criteria were applied to enhance the accuracy and reliability of outcome assessment. Patients treated solely in primary care or as outpatients likely had milder disease that did not require comprehensive documentation. Excluding prolonged hospitalizations (>30 days) helped eliminate cases with atypical or complicated clinical courses. Additionally, the removing records with missing or ambiguous antidiabetic medication data ensured a more precise evaluation of drug-related outcomes. Consequently, a total of 16,134 patients were enrolled, including 7082 who were prescribed DPP-4 inhibitors, and 9052 who were never prescribed these drugs (Figure 1).

The mean age of the patients was 68.23 ± 14.13 years in the DPP-4 inhibitor group and 69.94 ± 13.83 in the non-DPP-4 inhibitor group. Analysis of comorbidities revealed a slightly higher prevalence of hyperlipidemia, chronic renal failure, and ischemic heart disease in the DPP-4 inhibitor group. However, there was no statistically significant difference in the prevalence of chronic obstructive pulmonary disease between the two groups. Regarding outcomes, COVID-19 mortality was significantly lower in the DPP-4 inhibitor group (4.3%) compared to the non-DPP-4 inhibitor group (10.3%) The oxygen application rates were 26.9% in the DPP-4 inhibitor group and 25.0% in the non-DPP-4 inhibitor group, with no statistically significant difference. However, the proportion of patients requiring admission to the ICU due to worsening conditions was significantly lower in the DPP-4 inhibitor group (14.0%) than in the non-DPP-4 inhibitor group (16.6%) (Table 1).

### 3.2. Impact of DPP-4 Inhibitors in Angiotensin Receptor Blocker Users

We performed an additional subgroup analysis of patients using angiotensin receptor blocker (ARB), as we postulated that ARB use could affect this study due to the impact of DPP-4 inhibitors on the angiotensin receptor pathway (notably, no patients in our dataset were prescribed ACE inhibitors).

A total of 4214 patients were prescribed ARBs, of whom 2594 also received DPP-4 inhibitors and 1620 did not. In this ARB subgroup, mortality was significantly lower in the DPP-4 inhibitor group (3.6% vs. 7.2%). Oxygen prescription rates were 27.8 vs. 22.7% and ICU admission rates were 12.8% vs. 14.1%, respectively. Similarly, among patients not using ARB, mortality remained significantly lower in the DPP-4 inhibitor group (4.7% vs. 11.0%), and with ICU admission rates also reduced (14.0% vs. 17.4%) (Table 2).

### 3.3. Impact of DPP-4 Inhibitors in Insulin Users

Because DPP-4 inhibitors are less effective at lowering blood glucose than other diabetes medications, we were concerned that patients prescribed DPP-4 inhibitors might have had less severe diabetes., To address this potential bias, we examined whether COVID-19 severity differed based on insulin use. A total of 10,613 patients were taking insulin, of whom 3730 were prescribed DPP-4 inhibitors and 6234 were not. Mortality was statistically significantly lower in the DPP-4 inhibitor group (7.1% vs. 14.4%). Oxygen prescription rates were 40.2% vs. 31.6% and ICU care rates were 23.9% vs. 23.4%, respectively (Table 3).

In contrast, among the 6170 patients not prescribed insulin, there was no statistically significant difference in mortality between the DPP-4 inhibitor group (3352 patients) and the non-DPP-4 inhibitor group (2818 patients), with rates of 1.2% vs. 1.3%. Oxygen prescription rates were 12.1% vs. 10.4%, and ICU care at 3.0% vs. 1.7%, respectively (Table 3).

### 3.4. Impact of Sodium-Glucose Cotransporter 2 (SGLT2) Inhibitors in COVID-19 Patients

Additionally, we analyzed the impact of sodium-glucose cotransporter 2 (SGLT2) inhibitors on COVID-19-related outcomes. Among the 16,134 enrolled patients, 739 had been prescribed SGLT2 inhibitors. The 30-day mortality rate was significantly lower in the SGLT2 inhibitor group (3.2%) compared to non-users (6.3%, *p* = 0.001). However, the ICU admission rate was slightly higher among SGLT2 users than non-users (27.9% vs. 23.8%, *p* = 0.012).

### 3.5. Analysis of Mortality Effects-COX Regression, Kaplan-Meier Curves

Cox regression analysis was used to identify factors influencing mortality from COVID-19. Male sex and chronic renal failure were confirmed to be associated with a poorer prognosis. In multivariable Cox regression analysis adjusted for age, sex, and major comorbidities, DPP-4 inhibitor use was independently associated with a significantly lower risk of 30-day mortality (adjusted HR: 0.455; 95% CI: 0.414–0.499) (Table 4).

**Figure 2:**
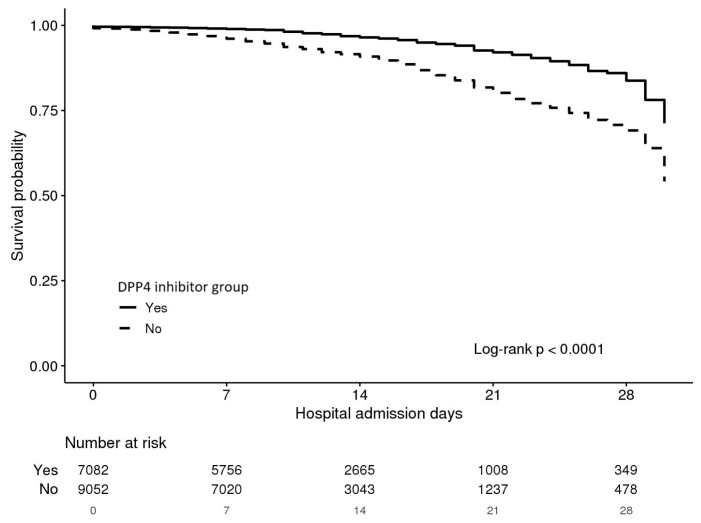
Kaplan-Meier Curve of Survival Rates by DPP-4 Inhibitor Use.

Figure 2 depicts Kaplan-Meier curves illustrating the effect of DPP-4 inhibitors on survival rates based on the length of hospital stay in diabetic patients infected with COVID-19. Compared to the control group, the survival rate was significantly higher in the treated with DPP-4 inhibitors (Figure 2).

### 3.6. Sensitivity Analyses Confirming Robustness of Findings

To confirm the robustness of our findings, we conducted sensitivity analyses using PSM. After 1:1 matching, 14,164 patients (7082 in each group) were included, with well-balanced baseline characteristics between DPP-4 inhibitor users and non-users (Supplementary Table S1). In the matched cohort, the 30-day mortality rate remained significantly lower in the DPP-4 inhibitor group compared to non-users (4.3% vs. 9.8%, *p* < 0.0001).

Consistently, IPTW-adjusted logistic regression confirmed a significant association between DPP-4 inhibitor use and reduced COVID-19-related mortality (OR: 0.30; 95% CI: 0.27–0.34; *p* < 0.001), reinforcing the robustness of our primary findings.

## 4. Discussion

This nationwide study provides evidence that DPP-4 inhibitor use in diabetic patients hospitalized with COVID-19 is associated with lower mortality rates. Our multivariable Cox regression analysis, adjusted for potential confounders including age, sex, and comorbidities, showed that DPP-4 inhibitor use was independently associated with reduced COVID-19-related mortality (adjusted HR: 0.455; 95% CI: 0.414–0.499). This adjustment suggests the association is unlikely to be solely due to baseline group differences. When we analyzed the observed phenomenon, we found that although the oxygen prescription rate was higher or unchanged in patients taking DPP-4 inhibitors, the proportion of patients requiring ICU care and the mortality rate were both lower. This suggests that DPP-4 inhibitors may help prevent the progression of COVID-19 to severe disease.

Results from previous studies on the use of DPP-4 inhibitors in COVID-19 patients have been mixed. A multicenter retrospective cohort study conducted in Saudi Arabia found no significant impact on mortality reduction or ICU treatment [16], whereas an observational meta-analysis reported that DPP-4 inhibitor use reduced COVID-19-related mortality by approximately 50% [17].

Recent studies suggest that DPP-4 inhibition may protect against COVID-19 by reducing viral entry and replication. While SARS-CoV-2 primarily uses ACE2, it can also interact with DPP-4, which shares residues with the viral spike protein, indicating that DPP-4 inhibitors may help limit viral penetration and load [18]. Beyond glucose metabolism, DPP-4 functions as an immunomodulator; its inhibition reduces inflammatory cytokines (e.g., IL-6, TNF-α) and improves endothelial function, potentially mitigating severe COVID-19–related inflammation and thromboembolic risk [14,19]. This dual effect, limiting viral entry and modulating immune responses, may underlie the observed mortality benefit, with DPP-4 inhibitors also possibly alleviating hypertension-related comorbidities in COVID-19 [20]. Taken together, these findings reinforce the potential therapeutic role of DPP-4 inhibitors in mitigating COVID-19 severity through both glycemic and pleiotropic mechanisms, warranting further mechanistic and prospective interventional studies.

Potential pharmacological interactions between DPP-4 inhibitors and medications commonly used in hospitalized COVID-19 patients warrant consideration. Corticosteroids, often administered for severe COVID-19, can exacerbate hyperglycemia and increase insulin requirements [21], potentially offsetting the glycemic benefits of DPP-4 inhibitors. Nonetheless, prior studies suggest that DPP-4 inhibitors can be safely combined with corticosteroids without significant adverse effects on glucose metabolism [22]. Similarly, antivirals such as remdesivir and nirmatrelvir/ritonavir do not appear to have clinically meaningful pharmacokinetic interactions with DPP-4 inhibitors, as they are metabolized through different hepatic pathways [23,24]. Regarding concomitant antidiabetic agents, DPP-4 inhibitors are often used alongside metformin or insulin. Our subgroup analysis showed that the mortality benefit of DPP-4 inhibitors persisted even among insulin users, suggesting potential additive or synergistic effects. Nevertheless, the absence of detailed data on the timing, dosage, and adherence to these concurrent medications in our claims-based database limits a more comprehensive assessment of drug interactions. This limitation highlights the need for future prospective studies incorporating granular pharmacologic data and pharmacodynamic monitoring.

Our findings align with those reported by Emral et al. in a nationwide Turkish study [19], which also found a significant association between DPP-4 inhibitor use and reduced COVID-19 mortality in patients with type 2 diabetes. Both studies used national electronic health records and applied propensity-matched or adjusted analyses to account for confounders. The Turkish study had a younger population (mean age: 62.8 years), whereas our Korean cohort was older (mean age: 69.2 years), reflecting Korea’s rapidly aging demographic. Despite this, the mortality rate in our cohort (7.7%) was comparable to that in Turkey (5.2%), suggesting a potential protective effect of DPP-4 inhibitors even in higher-risk populations. While ICU admission rates were not reported in the Turkish study, our study documented an ICU admission rate of 15.5%, providing additional insight into clinical severity among hospitalized Korean patients. Furthermore, our analysis incorporated additional severity indicators (e.g., oxygen therapy, ICU admission) and subgroup analyses (e.g., insulin and ARB use), offering a more granular understanding of disease progression and potential treatment interactions compared to the Turkish study [25].

Of note, our subgroup analysis of patients using insulin, who typically have more advanced diabetes and higher mortality risk, showed a significant survival benefit in those also taking DPP-4 inhibitors. This suggests that the observed effect is not merely due to better baseline glycemic control in the DPP-4 inhibitor group. Our findings align with previous observational studies suggesting a potential protective role of DPP-4 inhibitors in patients with type 2 diabetes mellitus and COVID-19, particularly among those receiving insulin therapy. Several retrospective cohort studies have demonstrated that adding a DPP-4 inhibitor to insulin treatment may be associated with a reduced mortality and severe outcomes. For instance, a multicenter observational study from Italy reported that in-hospital mortality among insulin-treated patients was markedly lower in those receiving sitagliptin along with insulin (18% vs. 37%) [26].

Our study is the first to examine the impact of DPP-4 inhibitors on COVID-19 mortality rates using nationwide big data from South Korea. We further strengthened the robustness of our findings by separately analyzing factors that could influence outcomes, such as insulin and ARB use. Additionally, this study is based on big data from the HIRA in South Korea, which covers the entire population of national health insurance enrollees, ensuring national-level representativeness without bias toward any particular group when analyzing COVID-19 inpatient data. Since 2020, in response to COVID-19, HIRA has operated a dedicated billing code and dataset for COVID-19 patients, enabling analysis of specialized information such as COVID-19 hospitalization status, severity (e.g., oxygen treatment, ventilator use, etc.), and treatment outcomes. This comprehensive and standardized national dataset is uniquely available in Korea, making large-scale, population-level analysis with detailed clinical outcomes virtually impossible in most other countries. This distinct advantage enhances the credibility and generalizability of our findings.

Despite these promising findings, our study has several limitations inherent to claims-based data. First, as an observational and retrospective study, causal inference remains limited, and residual confounding from unmeasured variables (e.g., BMI, smoking status, HbA1c, detailed cardiovascular history, or admission severity) cannot be ruled out despite adjustment for major comorbidities. Therefore, future prospective cohort studies and randomized controlled trials are warranted to confirm the protective association of DPP-4 inhibitors observed in our analysis and to further elucidate their potential role in improving outcomes among diabetic patients with COVID-19. Additionally, information on specific SARS-CoV-2 variants (e.g., Alpha, Delta, Omicron) was not available in the HIRA database and thus could not be accounted for in our analysis. Second, we were unable to distinguish between type 1 and type 2 diabetes, though the findings likely apply primarily to type 2 diabetes, which predominates in our cohort. Third, information on treatment duration, adherence, concomitant glucose-lowering agents, and individual DPP-4 inhibitor types or dosages were unavailable, limiting assessment of drug-specific effects and interactions. Fourth, we lacked information on whether DPP-4 inhibitors were initiated pre-hospital or in-hospital, precluding time-to-treatment analysis and raising the possibility that observed associations partly reflect pre-hospital exposure. Fifth, the absence of cause-specific mortality data prevented us from distinguishing COVID-19-related deaths from all-cause mortality. Sixth, vaccination status was not captured, which may have influenced outcomes given Korea’s high adult vaccination coverage (>85%) during the study period, however, this likely reduced imbalance between groups. Seventh, generalizability may be limited due to population-specific factors in Korea. Eighth, we acknowledge that oxygen therapy and ICU admission are imperfect proxies for disease severity, as they may vary by institutional protocols and healthcare capacity. Nevertheless, given the limitations of claims-based data, these indicators were the most feasible severity measures available, and our findings remained consistent even after adjusting for them in multivariable analyses. Future studies with more granular clinical data, treatment timing, vaccination status, and cause-of-death information are warranted to validate these findings. Lastly, although we used multivariable-adjusted Cox regression and subgroup analyses to address potential confounders, our study remains limited by the constraints of claims-based data and the lack of complementary methods such as propensity score matching or sensitivity analyses. These limitations may affect the robustness of our findings, highlighting the need for future studies employing advanced analytic techniques. Additionally, studies with more detailed clinical data, treatment timing, vaccination status, and cause-of-death information are needed to confirm these results.

In conclusion, our analysis of nationwide Korean data suggests that DPP-4 inhibitor use in diabetic patients hospitalized with COVID-19 may be associated with reduced disease severity and mortality. These findings contribute to the growing body of evidence supporting a potential protective role of DPP-4 inhibitors in the context of COVID-19. However, given the observational design, residual confounding cannot be excluded. Mechanistic pathways cited were based on prior literature without experimental validation, and clinically applicable measures such as number needed to treat were not reported, underscoring the need for prospective studies.

Given the retrospective design of our study, causal inference remains limited. To strengthen causal inference, future research should prioritize prospective randomized controlled trials specifically designed to evaluate variant-specific outcomes and incorporate detailed clinical and laboratory data (e.g., inflammatory markers, viral load, vaccination status). Additionally, large-scale international comparative studies could elucidate regional differences in outcomes, while mechanistic studies are needed to clarify the pleiotropic effects of DPP-4 inhibition in COVID-19.

## Figures and Tables

**Table 1:** Baseline characteristics of the study population.

	Total (n = 16,134)	Non-DPP-4 Inhibitor Group (n = 9052)	DPP-4 Inhibitor Group (n = 7082)	*p*-Value
Age (years)	69.19 ± 13.99	69.94 ± 13.83	68.23 ± 14.13	<0.0001
Sex (Male)	8823 (54.7)	4806 (53.1)	4017 (56.7)	<0.0001
Length of Stay (days)	12.08 ± 7.25	11.81 ± 7.26	12.42 ± 7.23	<0.0001
Death	1237 (7.7)	933 (10.3)	304 (4.3)	<0.0001
Hypertension	4341 (26.9)	1669 (18.4)	2672 (37.7)	<0.0001
Hyperlipidemia	5378 (33.3)	2257 (24.9)	3121 (44.1)	<0.0001
Chronic Kidney Disease	1209 (7.5)	486 (5.4)	723 (10.2)	<0.0001
Chronic Obstructive Pulmonary Disease	895 (5.5)	511 (5.6)	384 (5.4)	0.5392
Ischemic Heart Disease	1682 (10.4)	865 (9.6)	817 (11.5)	<0.0001
Stroke	0 *	0	0	
Congestive Heart Failure	1 (0.0)	1 (0.0)	0	>0.999
End-Stage Renal Disease	0	0	0	
Oxygen demand	4166 (25.8)	2261 (25.0)	1905 (26.9)	0.0057
Intensive care unit admit	2496 (15.5)	1503 (16.6)	993 (14.0)	<0.0001

* Values listed as ‘0’ for stroke, congestive heart failure, and end-stage renal disease indicate the absence of these comorbidities in the study cohort, rather than missing or unrecorded data.

**Table 2:** Impact of DPP-4 inhibitor in angiotensin receptor blocker users.

		Angiotensin Receptor Blocker (−)				Angiotensin Receptor Blocker (+)		
	Total (n = 11,920)	Non-DPP-4 Inhibitor Group (n = 7432)	DPP-4 Inhibitor Group (n = 4488)	*p*-Value	Total (n = 4214)	Non-DPP-4 Inhibitor Group (n = 1620)	DPP-4 Inhibitor Group (n = 2594)	*p*-Value
Age (years)	68.4 ± 14.3	69.3 ± 14.00	66.9 ± 14.8	<0.0001	71.4 ± 12.7	72.9 ± 12.8	70.5 ± 12.6	<0.0001
Sex (Male)	6633 (55.6)	4003 (53.9)	2630 (58.6)	<0.0001	2190 (52.0)	803 (49.6)	1387 (53.5)	0.014
Length of Stay (days)	11.8 ± 7.0	11.6 ± 7.1	12.2 ± 7.0	<0.0001	12.9 ± 7.8	13.0 ± 8.0	12.8 ± 7.6	0.416
Death	1027 (8.6)	817 (11.0)	210 (4.7)	<0.0001	210 (5.0)	116 (7.2)	94 (3.6)	<0.0001
Hypertension	127 (1.1)	49 (0.7)	78 (1.7)	0.056	4214 (100)	1620 (100)	2594 (100)	>0.999
Hyperlipidemia	3135 (26.3)	1538 (20.7)	1597 (35.6)	<0.0001	2243 (53.2)	719 (44.4)	1524 (58.8)	<0.0001
Chronic Kidney Disease	702 (5.9)	340 (4.6)	362 (8.1)	<0.0001	507 (12.0)	146 (9.0)	361 (13.9)	<0.0001
Chronic Obstructive Pulmonary Disease	671 (5.6)	444 (6.0)	227 (5.1)	0.0355	224 (5.3)	67 (4.1)	157 (6.1)	0.007
Ischemic Heart Disease	1055 (8.9)	647 (8.7)	408 (9.1)	0.4730	627 (14.9)	218 (13.5)	409 (15.8)	0.0404
Stroke	0 *	0	0		0	0	0	
Congestive Heart Failure	0	0	0		1 (0.0)	1 (0.1)	0 (0.0)	>0.999
End-Stage Renal Disease	0	0	0		0	0	0	
Oxygen demand	3078 (25.8)	1894 (25.5)	1184 (26.4)	0.2782	1088 (25.8)	367 (22.7)	721 (27.8)	<0.0001
Intensive care unit admit	1923 (16.1)	1296 (17.4)	627 (14.0)	<0.0001	573 (13.6)	207 (12.8)	366 (14.1)	0.2199

* Values are presented as number (percentage) or mean ± standard deviation. Values listed as ‘0’ for stroke, congestive heart failure, and end-stage renal disease indicate the absence of these comorbidities in the study cohort, rather than missing or unrecorded data.

**Table 3:** Impact of DPP-4 inhibitor in insulin users.

		Insulin (−)				Insulin (+)		
	Total (n = 6170)	Non-DPP-4 Inhibitor Group (n = 2818)	DPP-4 Inhibitor Group (n = 3352)	*p*-Value	Total (n = 9964)	Non-DPP-4 Inhibitor Group (n = 6234)	DPP-4 Inhibitor Group (n = 3730)	*p*-Value
Age (years)	69.2 ± 14.6	70.6 ± 14.5	68.1 ± 14.5	<0.0001	69.2 ± 13.6	69.7 ± 13.5	68.3 ± 13.8	<0.0001
Sex (Male)	3191 (51.7)	1338 (47.5)	1853 (55.3)	<0.0001	5632 (56.5)	3468 (55.6)	2164 (58.0)	0.0201
Length of Stay (days)	11.8 ± 7.3	12.5 ± 7.8	11.2 ± 6.8	<0.0001	12.2 ± 7.2	11.5 ± 7.0	13.5 ± 7.4	<0.0001
Death	77 (1.2)	37 (1.3)	40 (1.2)	0.6732	1160 (11.6)	896 (14.4)	264 (7.1)	<0.0001
Hypertension	2047 (33.2)	804 (28.5)	1243 (37.1)	<0.0001	2294 (23.0)	865 (13.9)	1429 (38.3)	<0.0001
Hyperlipidemia	2271 (36.8)	827 (29.3)	1444 (43.1)	<0.0001	3107 (31.2)	1430 (22.9)	1677 (45.0)	<0.0001
Chronic Kidney Disease	314 (5.1)	53 (1.9)	261 (7.8)	<0.0001	895 (9.0)	433 (6.9)	462 (12.4)	<0.0001
Chronic Obstructive Pulmonary Disease	238 (3.9)	100 (3.5)	138 (431)	0.2482	657 (6.6)	411 (6.6)	246 (6.6)	0.9964
Ischemic Heart Disease	574 (9.3)	233 (8.3)	341 (10.2)	0.0001	1108 (11.1)	632 (10.1)	476 (12.8)	<0.0001
Stroke	0 *	0	0		0	0	0	
Congestive Heart Failure	0	0	0		1 (0.0)	1 (0.0)	0 (0.0)	>0.999
End-Stage Renal Disease	0	0	0		0	0	0	
Oxygen demand	699 (11.3)	292 (10.4)	407 (12.1)	0.0280	3467 (34.8)	1969 (31.6)	1498 (40.2)	<0.0001
Intensive care unit admit	149 (2.4)	47 (1.7)	102 (3.0)	0.0005	2347 (23.6)	1456 (23.4)	891 (23.9)	0.545

* Values are presented as number (percentage) or mean ± standard deviation. Values listed as ‘0’ for stroke, congestive heart failure, and end-stage renal disease indicate the absence of these comorbidities in the study cohort, rather than missing or unrecorded data.

**Table 4:** Cox Regression for Mortality in Enrolled Patients.

	Univariable		Multivariable-Stepwise	
	Hazard Ratio (95% CI)	*p* Value	Hazard Ratio (95% CI)	*p* Value
Age	1.051 (1.036–1.047)	<0.0001		
Sex (Male)	1.188 (1.057–1.335)	0.0037	1.166 (1.071–1.267)	0.0003
Hypertension	0.544 (0.468–0.632)	<0.0001	0.555 (0.498–0.617)	<0.0001
Hyperlipidemia	0.748 (0.654–0.855)	<0.0001	0.744 (0.675–0.819)	<0.0001
Chronic kidney diseases	1.514 (1.263–1.814)	<0.0001	1.519 (1.336–1.727)	<0.0001
Chronic obstructive pulmonary diseases	1.091 (0.87–1.368)	0.451		
Ischemic heart disease	1.158 (0.974–1.377)	0.097	1.171 (1.034–1.326)	<0.0001
Stroke	-	-		
Congestive heart failure	-	-		
End-stage renal diseases	-	-		
DPP-4 inhibitor	0.455 (0.397–0.521)	<0.0001	0.455 (0.414–0.499)	<0.0001

## Data Availability

The datasets used and/or analysed during the current study available from the corresponding author on reasonable request.

## Supplementary Materials

The following supporting information can be downloaded at: https://www.mdpi.com/article/10.3390/jcm14165815/s1.
