# Supplementary material for: Association of Dipeptidyl Peptidase-4 Inhibitor Use with COVID-19 Mortality in Diabetic Patients: A Nationwide Cohort Study in Korea
Source: J Clin Med. 2025 Aug 17;14(16):5815. doi: 10.3390/jcm14165815 (PMC12387907; doi:10.3390/jcm14165815)
Supplement: Supplementary file 1 [file jcm-14-05815-s001.zip › jcm-3765857-supplementary.pdf]

	Propensity Score Matching			
	**Total (n = 14,164)**	**non-DPP4 inhibitor group (n = 7082)**	**DPP-4 inhibitor group (n = 7082)**	P-value
Age (years)	68.3 ± 14.0	68.3 ± 14.0	68.23 ± 14.13	0.6719
Sex (Male)	8028 (56.7)	4011 (56.6)	4017 (56.7)	0.9190
Length of Stay (days)	12.1 ± 7.2	11.7 ± 7.1	12.42 ± 7.23	<0.0001
Death	996 (7.0)	692 (9.8)	304 (4.3)	<0.0001
Hypertension	3948 (27.9)	1276 (18.0)	2672 (37.7)	<0.0001
Hyperlipidemia	4892 (34.5)	1771 (25.0)	3121 (44.1)	<0.0001
Chronic Kidney Disease	1097 (7.7)	374 (5.3)	723 (10.2)	<0.0001
Chronic Obstructive Pulmonary Disease	791 (5.6)	407 (5.7)	384 (5.4)	0.4000
Ischemic Heart Disease	1488 (10.5)	671 (9.5)	817 (11.5)	<0.0001
Stroke	0 (0.0)	0 (0.0)	0	-
Congestive Heart Failure	0 (0.0)	0 (0.0)	0	-
End-Stage Renal Disease	3703 (26.1)	1798 (25.4)	0	0.0408
Oxygen demand	2196 (15.5)	1203 (17.0)	1905 (26.9)	<0.0001
